# Static magnetic field regulates *Arabidopsis* root growth via auxin signaling

**DOI:** 10.1038/s41598-019-50970-y

**Published:** 2019-10-07

**Authors:** Yue Jin, Wei Guo, Xupeng Hu, Mengmeng Liu, Xiang Xu, Fenhong Hu, Yiheng Lan, Chenkai Lv, Yanwen Fang, Mengyu Liu, Tieliu Shi, Shisong Ma, Zhicai Fang, Jirong Huang

**Affiliations:** 1 Shanghai Key Laboratory of Plant Molecular Sciences, College of Life Sciences, Shanghai Normal University, Shanghai, 200234 China; 2 Institute of Plant Physiology and Ecology, Chinese Academy of Sciences, Shanghai, 200032 China; 3 Shanghai Key Laboratory of Regulatory Biology, School of Life Sciences, East China Normal University, Shanghai, 200241 China; 4 Heye Health Industrial Research Institute of Zhejiang Heye Health Technology, Anji, Zhejiang 313300 China; 5 School of Life Sciences, University of Science and Technology of China, Hefei, 230026 China

**Keywords:** Plant sciences, Plant physiology

## Abstract

Static magnetic field (SMF) plays important roles in biological processes of many living organisms. In plants, however, biological significance of SMF and molecular mechanisms underlying SMF action remain largely unknown. To address these questions, we treated *Arabidopsis* young seedlings with different SMF intensities and directions. Magnetic direction from the north to south pole was adjusted in parallel (N0) with, opposite (N180) and perpendicular to the gravity vector. We discovered that root growth is significantly inhanced by 600 mT treatments except for N180, but not by any 300 mT treatments. N0 treatments lead to more active cell division of the meristem, and higher auxin content that is regulated by coordinated expression of PIN3 and AUX1 in root tips. Consistently, N0-promoted root growth disappears in *pin3* and *aux1* mutants. Transcriptomic and gene ontology analyses revealed that in roots 85% of the total genes significantly down-regulated by N0 compared to untreatment are enriched in plastid biological processes, such as metabolism and chloroplast development. Lastly, no difference in root length is observed between N0-treated and untreated roots of the double cryptochrome mutant *cry1 cry2*. Taken together, our data suggest that SMF-regulated root growth is mediated by CRY and auxin signaling pathways in *Arabidopsis*.

## Introduction

All organisms living on the earth are inevitably subject to an action of the geomagnetic field (GMF), which can be represented by a three-dimensional vector called intensity, inclination and declination. The intensity of GMF at the earth’s surface ranges from 25 to 65 microteslas (μT) and gradually decreases from the poles to the equator^1^. It is well known that many organisms can utilize the weak GMF for their environmental adaptations. For example, magnetotactic bacteria fix their best living position with an optimal oxygen concentration by perceiving the cues of GMF^2^, animals can use GMF information to accurately navigate during migration, nest or burrow^3^, *Arabidopsis* plants grow more slowly and flower later in a near-zero magnetic field condition than in GMF^4–8^. Interestingly, reversing the GMF polarity greatly inhibits growth of hypocotyls and roots, and modulates gene expression, suggesting that GMF might have been a factor contributing to plant evolution over geological timescales^9^. Unfortunately, the molecular mechanism by which living organisms sense GMF and transduce the signal in a cell remains largely unknown.

Plant responses to the static magnetic field (SMF) have been observed in a wide range of species, and were well summarized in several review papers^10,11^. Seed germination rates were frequently improved when seeds were pretreated with a moderate intensity of SMF^12–15^. Stem length of rice (*Oryza sativa*) treated by 125 mT or 250 mT for ten days was significantly enhanced compared to controls^16^. Furthermore, strong SMF treatment significantly altered expression of many genes in *Arabidopsis* seedlings^17^.

Cryptochromes (CRY) are highly conserved blue-light-absorbing photoreceptors, and have been suggested to sense magnetic stimuli via a CRY-based radical-pair hypothesis^18–21^. The employment of *Arabidopsis* as a model system has greatly accelerated our understanding of plant magnetoperception. Recently, *Arabidopsis* CRY-mediated magnetic sensitivity was reported during flavin reoxidation in the dark^22^, and could be modulated by the intensity and orientation of the local GMF^23^. Under the near-null magnetic field (NNMF, ≤40 nT) condition, *Arabidopsis* CRYs were shown to be involved in delayed flowering via modulating GA and auxin signaling^5–8^, and in changes of gene expression^24,25^.

Plant root growth is maintained by the meristem, in which the stem cell niche (SCN), comprising the mitotically inactive quiescent center (QC) and its surrounding stem cells, provides the source of all root cell types. Auxin has been considered as a central regulator in controlling SCN formation during early root development^26^. Auxin regulates primary root elongation and meristem size by manipulating cell division, expansion, and differentiation^27,28^. In roots, auxin distributes in a basipetal manner with the maximum around the QC, which is maintained by a polar auxin transport (PAT) system composed of auxin efflux transporters PINFORMED (PIN) proteins and influx carriers such as AUX1. In roots, PIN1, PIN2, PIN3 and PIN7 are the main efflux transporters^29–31^, while AUX1 acts as the main influx carrier^32^. Under natural growth conditions, auxin signaling is integrated with various environmental cues and with other hormonal signal transduction pathways as well^33^. In this study, we used *Arabidopsis* as a model system to investigate effects of SMF on seedling growth, and found that SMF promotes root growth in an intensity- and direction-dependent manner via increasing auxin concentrations at the root tip, providing a new system to elucidate molecular mechanisms of plant responses to SMF in the future.

## Results

### The angle between magnetic and gravity directions affects root growth in 600 mT SMF

**Figure 1:**
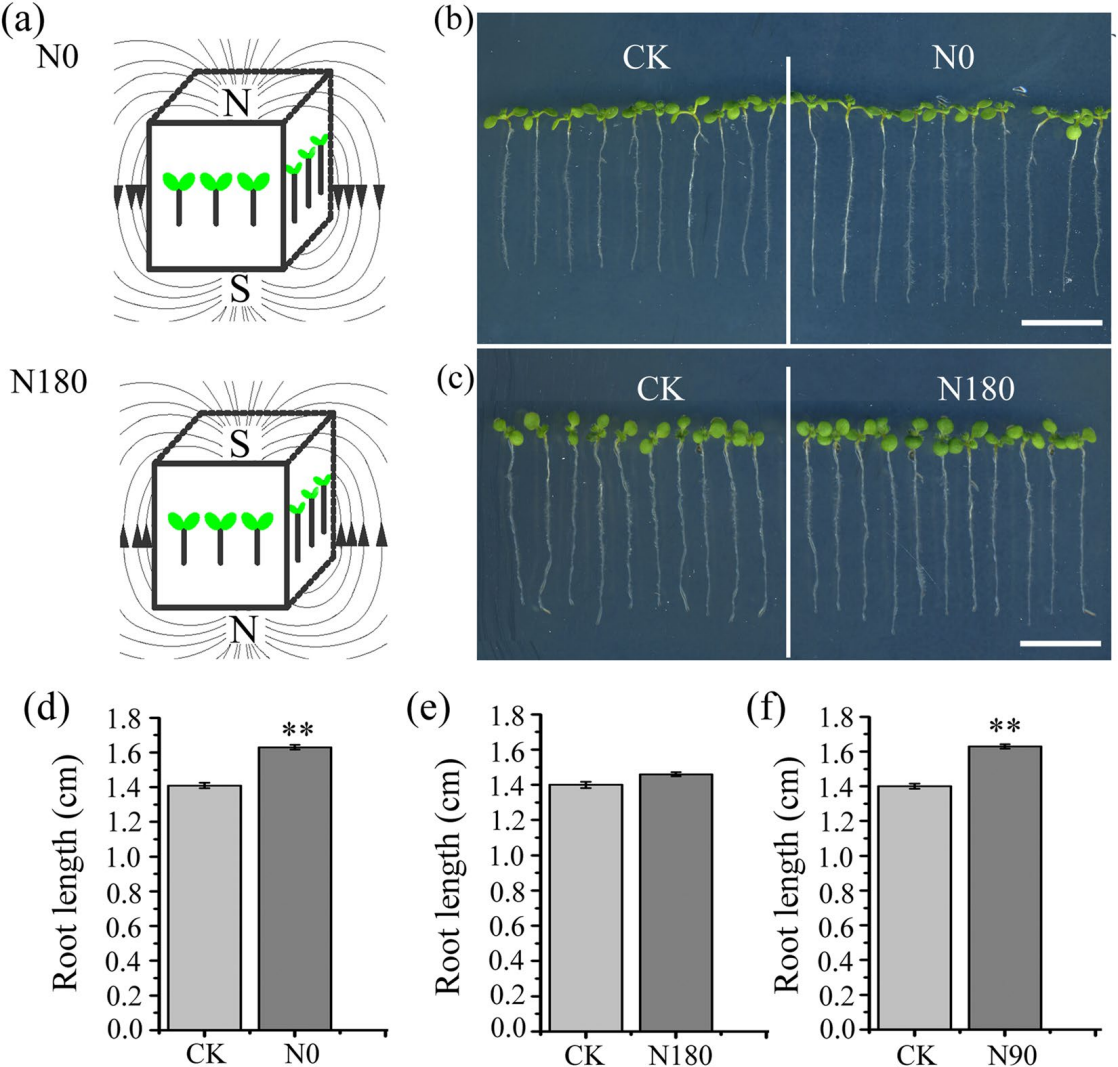
Effects of 600 mT SMF directions on root growth. (**a**) A diagram shows examples of N0 and N180 treatments, in which the SMF directions are in parallel with and opposite to that of root growth, respectively. The arrow indicates the direction of SMF from the north (N) to south (S) pole. (**b,c**) The representative 7-day-old seedlings treated with N0 (**b**) or N180 (**c**) and without 600 mT SMF (CK). Scale bar, 1 cm. (**d–f**) Quantitative analysis of the primary root length of seedlings grown in N0, N180 and N90 conditions. N0, N180 and N90 mean that the SMF direction is adjusted in the same direction as, in opposite, or perpendicular to the gravity vector, respectively. The data are shown means ± SE (n > 60). **Indicate significant differences at *P* < 0.01 (Student *t*-test). At least three independent biological repeats were conducted.

To investigate whether plant growth and development are affected by moderate SMF intensity (between 1 mT and 1 T)^34^, we vertically grew *Arabidopsis* seedlings in petri dishes that were closely attached to the side of a magnet, which is a cube with each side length of 10 cm and produces around 600 mT magnetic field at the surface of the north (N) and south (S) poles. Considering that petri dishes contained the solid medium and the roots actually grew about 5 mm away from the magnet surface, we measured magnetic fields 5 mm away from the magnet surface. Magnetic intensities at the side in parallel with the direction of magnetization ranged from 580 mT to 420 mT with the highest close to the poles and the lowest at the middle between the two poles, while at the side of the N or S pole ranged from 550 mT to 460 mT with the highest at the edge and the lowest at the center. At the beginning, we observed significant variations in root length of seedlings collected from different magnet-treated batches, but always the same from those of the corresponding control. We inferred that root growth might be affected by the differences of the angle between directions of SMF (from the north- to the south-pole) and root growth (gravity). To test this hypothesis, we designed three angles between the gravity and SMF directions, namely the SMF direction is in parallel (N0) with, opposite (N180), perpendicular (N90) to the gravity vector (Figs 1a and S1). As shown in Fig. 1b–f, N0 and N90 treatments significantly enhanced root length up to 10.8% and 8.6%, respectively, compared to their corresponding controls, whereas N180 treatment had no significant effect on root growth. We further tested other two types of N90 treatments, namely petri dishes were putted to the north (N90N) or south (N90S) pole (Supplementary Fig. S1a). The results showed that N90N and N90S also significantly promoted root growth (Supplementary Fig. S1). However, no apparent changes were observed in cotyledons and hypocotyls of the SMF-treated seedlings (Supplementary Fig. S2). In addition, we did not find significant effects of 300 mT SMF on seedling growth, compared to the control (Supplementary Fig. S3). These results suggest that responses of *Arabidopsis* young seedlings to SMF stimuli are influenced not only by its strength and orientation, but also by different tissues.

### SMF-stimulated root growth is due to an increase in cell number

**Figure 2:**
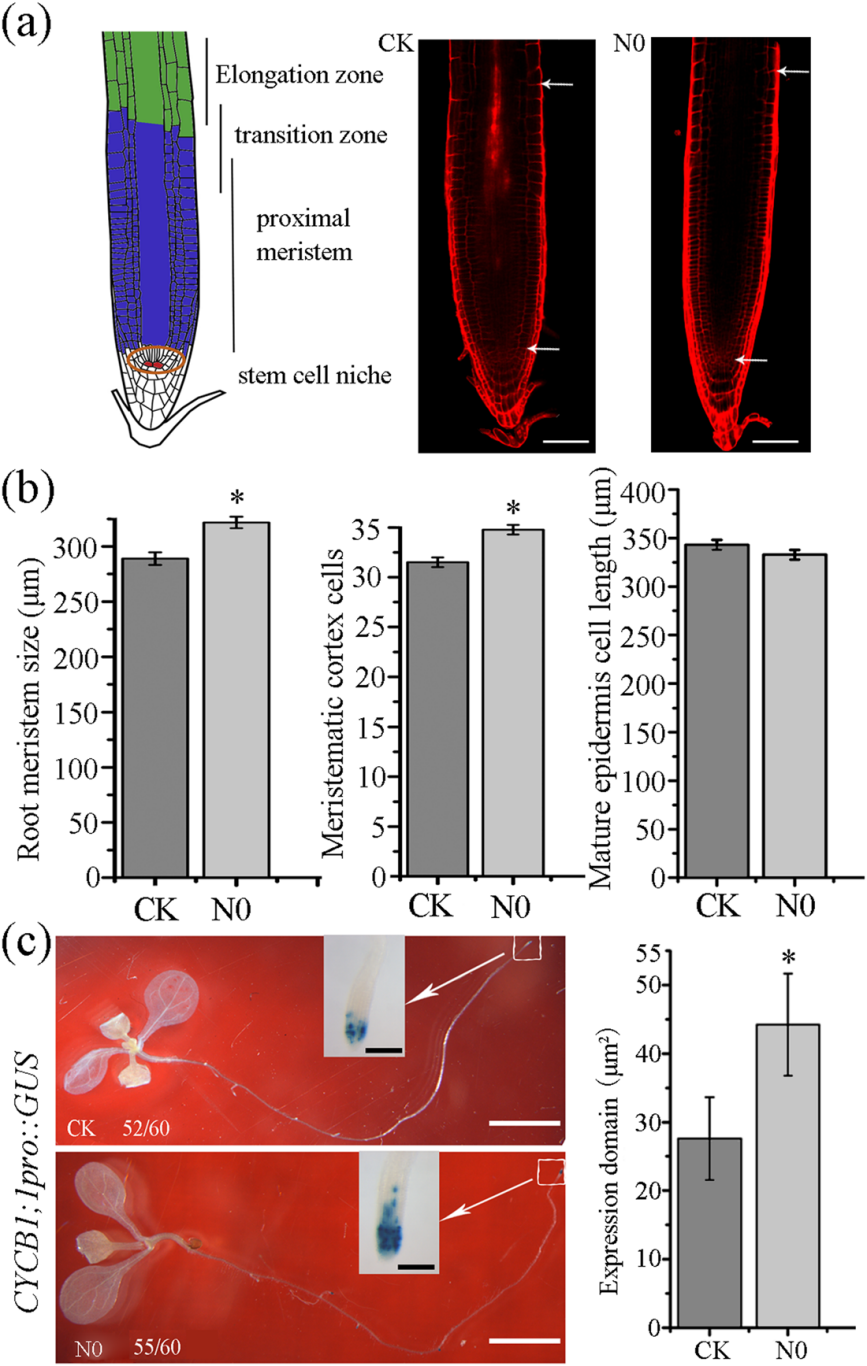
SMF-stimulated root growth is due to an increase in cell number. (**a**) A diagram shows an example of root cell distribution and a representative root meristem marked with two arrows from 7-day-old seedlings grown in N0 or in GMF (CK). Scale bar, 50 μm. (**b**) Quantification of root meristem size, meristematic cortex cell number and mature epidermis cell length of seedlings shown in (**a**). The data are presented means ± SE (n > 60). **Indicate significant differences at *P* < 0.01 (Student *t*-test). At least three independent biological experiments were performed. (**c**) GUS staining and quantification of a 10-day‐old transgenic seedling expressing *CYCB1;1pro::GUS* under the N0 or control condition. Scale bar, 0.25 cm. The ratio in the picture indicates the number of roots with the same GUS-staining as shown in the picture to the total examined. The inserted picture is the magnified root tip, Scale bar, 100 μm. Right panel was quantification of CYCB1;1pro::GUS in root tips.

The rate of root growth is determined by cell number, cell length, or both. To dissect roles of these components in the SMF-promoted root growth, we used N0-treated seedlings for further study. The root meristem zone was defined the region from the quient cell (QC) to the first elongated cortica cell (Fig. 2a). Our results showed that the meristem size of N0-treated seedlings was 316.88 μm in length, which is 9.66% longer than that of the SMF-untreated seedlings (288.97 μm) (Fig. 2a,b). Further analysis revealed that the N0-treated root meristem zone had more number of cortex cells than the control (Fig. 2b). However, SMF treatment had no effect on cell length in the mature zone (Fig. 2b). These results suggest that SMF-promoted root growth is attributed to the increased cell number. We then examined SMF effect on cell division using the transgenic line expressing the β-glucuronidase (GUS) driven by the native promoter of *CyclinB1;1*, which is a broadly used marker to indicate the G2 to M phase of the cell cycle^35^. As shown in Fig. 2c, the GUS expression domain was significantly larger in the SMF-treated root meristem than in the corresponding control. Consistently, quantification of GUS staining area showed that the total staining area in N0-treated roots was 54% higher than that in the control. Taken together, these results suggest that SMF promotes root growth via enhancing cell division.

### Transcriptomic changes in N0- and N180-treated roots and leaves

**Figure 3:**
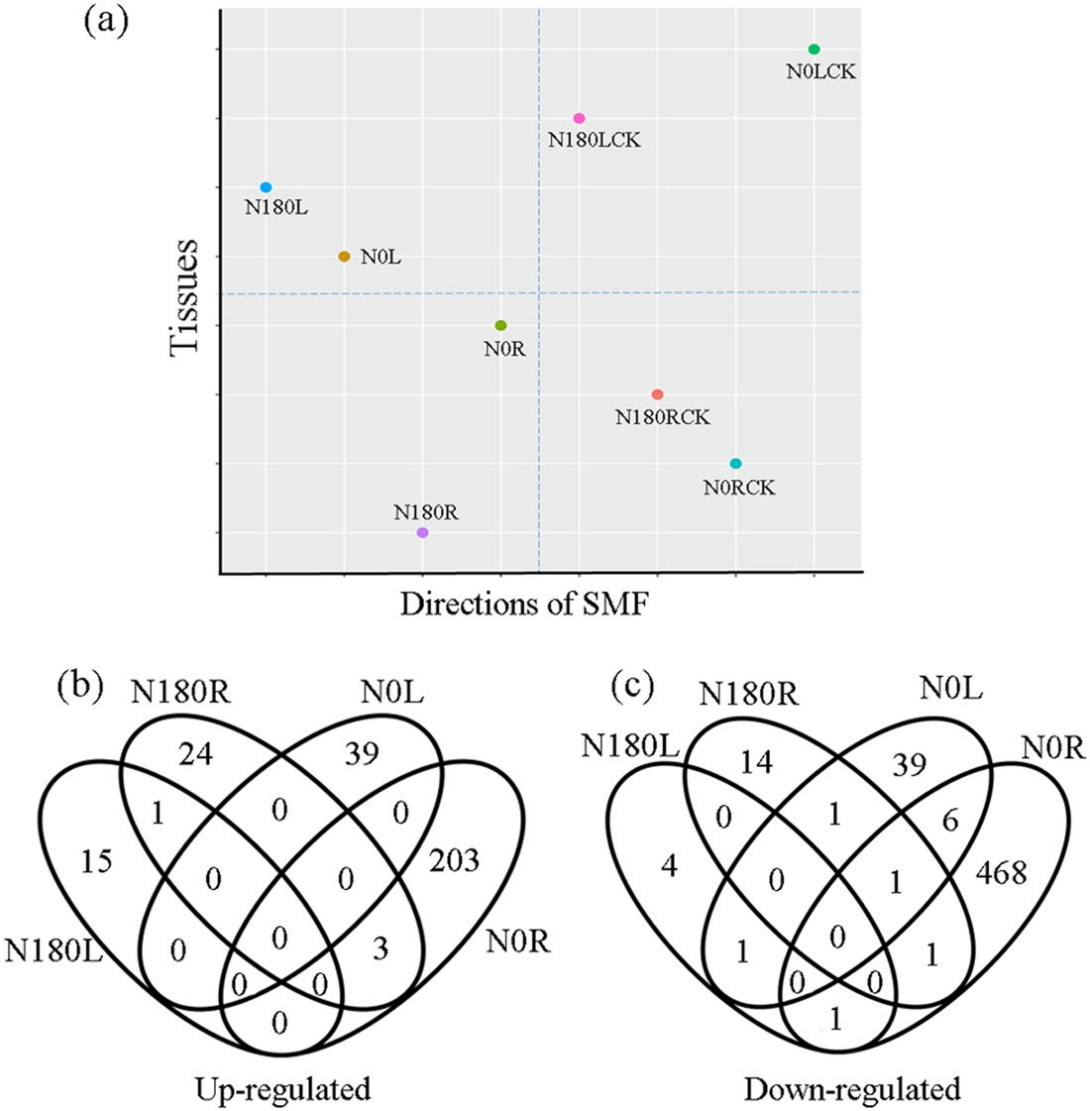
Transcriptomic analysis of roots and leaves sampled from 7-day-old N0- and N180-treated seedlings. (**a**) Principal component analysis of the samples using transcriptomic data averaged from three independent biological replicates. The first two principal components are shown. R, roots; L, leaves. (**b, c**) Venn diagrams for upregulated (**b**) and downregulated (**c**) genes by SMF treatments in roots and leaves.

To better understand molecular mechanism by which seedling growth is differentially affected by SMF treatments, we investigated global gene expression in the roots (R) and leaves (L) using RNA sequencing (RNA-Seq). The general information on the RNA-Seq data was summarized in Supplementary Tables S1 and S2. Correlation analysis showed that Pearson’s coefficients between biological replicates ranged from 0.92 to 0.99 (Supplementary Table S3), indicating that RNA-Seq data derived from three biological repeats are stable at the genome-wide level. Consistently, principal component analysis (PCA) revealed that the key factors that classified the samples into different groups were SMF treatments (N0 and N180) and tissues (roots and leaves) (Fig. 3a).

**Figure 4:**
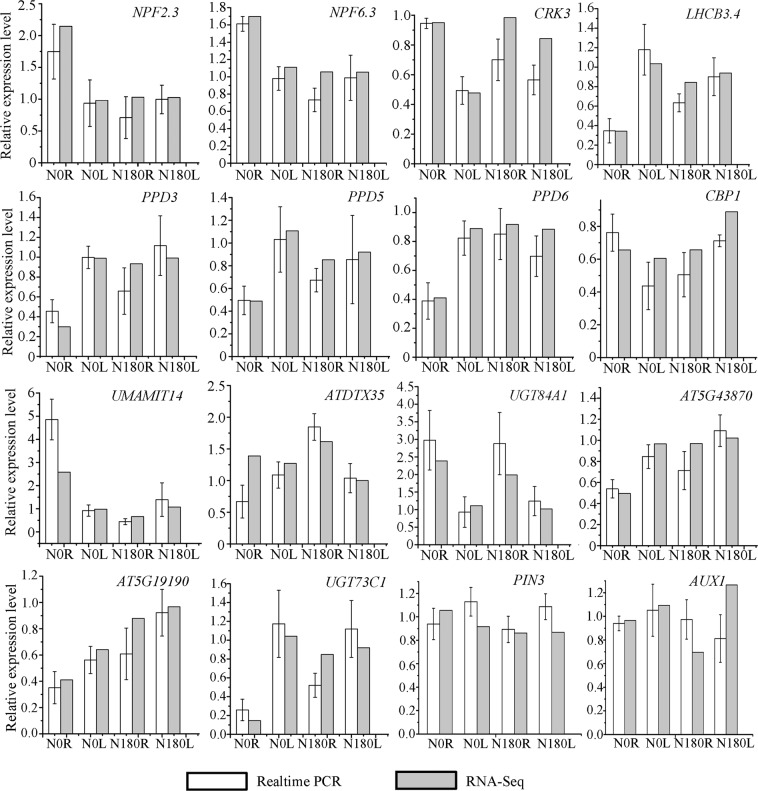
Comparison of RNA-Seq and qRT-PCR results. *NPF2.3* (AT3G45680) and *NPF6.3* (AT1G12110) encode an NRT1.1 and Peptide transporter Family protein; *CRK3* (AT1G70530), Cysteine-Rich receptor-like protein Kinase 3; *LHCB3.4* (AT2G40100), Light-Harvesting Chlorophyll B-Binding family protein; *PPD3* (AT5G27390), *PPD5* (AT5G11450) and *PPD6* (AT3G56650), a PsbP-Domain protein; *CBP1* (AT2G15890), CCG-Binding Protein 1; *UMAMIT14* (AT2G39510), Usually Multiple Acids Move In and out Transporters 14; *ATDTX35* (AT4G25640), Detoxifying Efflux Carrier 35; *UGT84A1* (AT4G15480), UDP-glucose Glucosyltransferase activity; AT5G43870, an auxin canalization protein. AT5G19190, a hypothetical protein; *UGT73C1* (AT2G36750), a protein that have sinapic acid: UDP-glucose glucosyltransferase activity; *PIN3*, PINFORMED (PIN) protein 3; *AUX1*, auxin influx carrier. AT5G19190, a hypothetical protein; *UGT73C1* (AT2G36750), a protein that have sinapic acid: UDP-glucose glucosyltransferase activity.

DESeq2 analysis was employed to calculate gene expression levels in the SMF-treated tissues (L, leaves; R, roots) relative to the corresponding untreated tissues (Supplementary Tables S4 and S5), and yielded 22, 45, 87 and 683 differentially expressed genes (DEGs) with fold change ≥1.5 (*p* < 0.05) for N180L, N180R, N0L, and N0R samples, respectively (Fig. 3b,c, Supplementary Table S5). These results showed that the number of DEGs identified from N0-treated roots (N0R) were 15.2-fold of that from N180R. Among these DEGs, the most up-regulated (At5G59680, 3.46-fold) and down-regulated (At5G12020, 6.96-fold) genes were present in N0R. In addition, more DEGs were found in roots than in leaves under both N0 and N180 conditions. These data suggest that N0 treatment has a broader effect on gene expression than N180 treatment, and that the root is more sensitive to SMF than the leaf. We also randomly selected 16 genes from the four groups of DEGs to validate the data of RNA-Seq analysis via quantitative PCR (qPCR). In general, our qRT–PCR results were very similar to those of RNA-Seq analysis (Fig. 4).

To see whether different SMF orientations induce common or diverse transcriptional responses, we screened for genes that were present in the all four or only in some DEG groups. Our results showed that there was no common DEG in the four groups, but a few genes existed in three or two groups (Fig. 3 and Supplementary Table S6), indicative of a specificity of different tissues in responses to SMF directions. Only one gene At3g23080, which encodes a polyketide cyclase/dehydrase, was down-regulated in N0L, N0R and N180R samples. The N0-treated roots and leaves share the highest DEGs, 9 genes, in which 7 genes were down-regulated, one up-regulated and one opposite, whereas only one common DEG (SHM7, AT1G36370) for N180R and N180L. This result indicates that the effect of N0 on gene expression is greater than that of N180. There are three up-regulated and one down-regulated common genes between N0R and N180R samples (Supplementary Table S6). Interestingly, all three up-regulated genes (UGT84A1, UGT84A2 and UGT78D4) in N0R and N180R encode UDP-glucosyltransferase (UGT) (Supplementary Tables S6 and S7). UGTs glycosylate a broad range of acceptor molecules, including plant hormones and all major classes of plant secondary metabolites^36,37^. Recently, UGT84A2 was reported to be the indole-3-butyric acid (IBA, an auxin precursor)-specific glycosyltransferase, and its ectopic expression caused an increase in the IBA level and an obvious delay in flowering in *Arabidopsis*^38^. Besides catalyzing the glycosylation of IBA, UGT84A2 has also been demonstrated to glycosylate sinapic acid, which is used for sinapate metabolism at the early stage of seedling growth and development^39,40^. Thus, whether UGT84A2 plays a role in IBA-IAA conversion or sipanate biosynthesis or both under our experimental conditions remains unclear. Only one gene related chloroplast function (LHCB2.4) was common for N0L and N180L. In addition, we found two and eight UGTs whose expression levels were significantly altered only in N180R and N0R, respectively (Supplementary Table S7). These results indicate that glycosylation may be involved in various biological processes in response to SMF. In the future, it is worth investigating the functions of these UGTs in plant responses to SMF.

### Overview of biological processes regulated by SMF in roots and leaves

**Figure 5:**
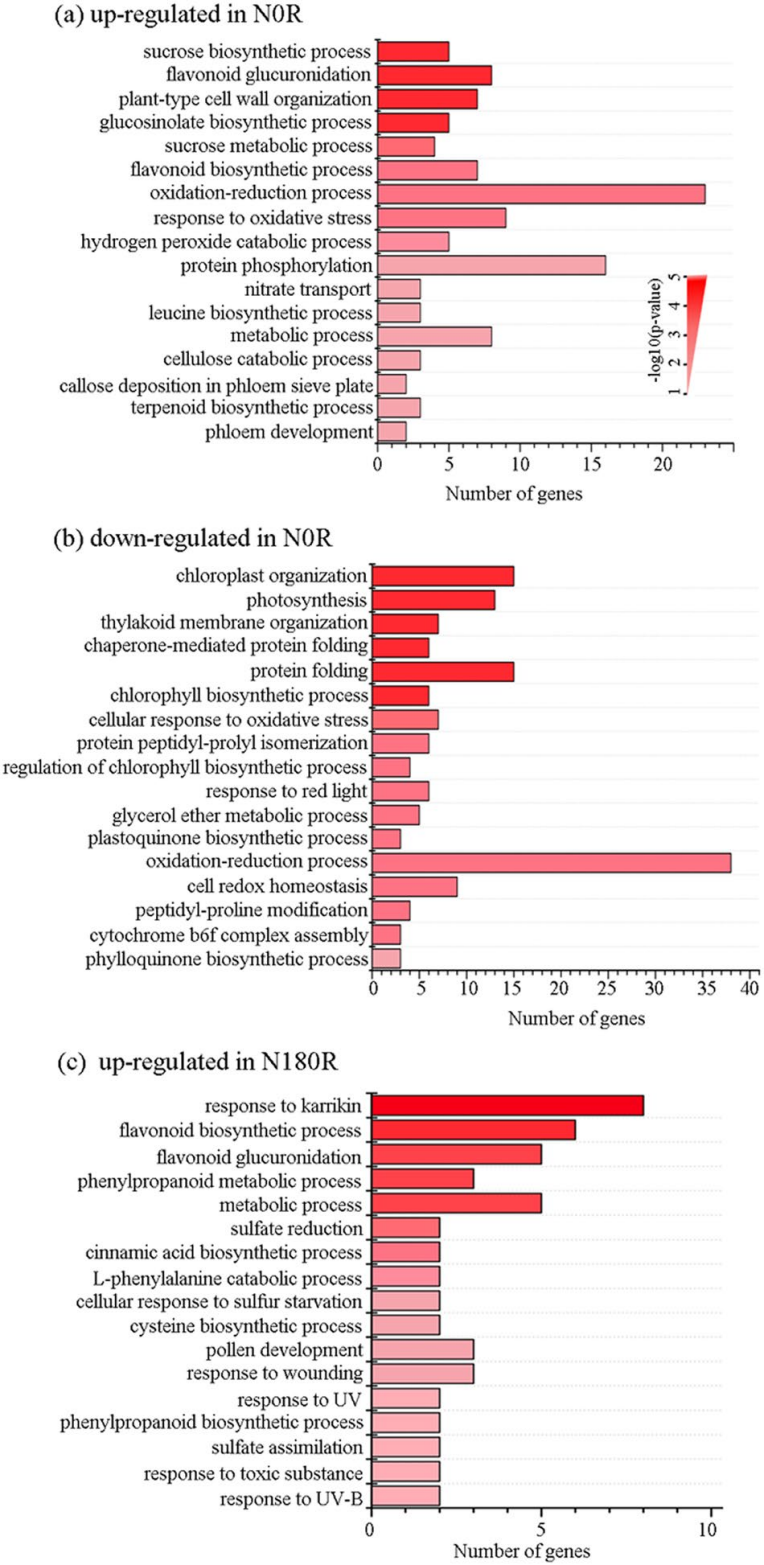
GO analysis of biological processes affected by SMF in roots and leaves based on DEGs with fold change ≥1.5 (*p* < 0.05) from RNA-Seq analysis. Shown are genes upregulated or downregulated by SMF across different tissues and various directions of SMF. Tissues name: N0R for roots under the N0 treatment, N180R for roots under the N180 treatment.

To interpret functions of the SMF-regulated genes in roots and leaves, we performed gene ontology (GO) enrichment analysis. In both SMF-treated roots (N0 and N180), up-regulated DEGs were enriched in the metabolic process, such as the flavonoid biosynthetic pathway (GO: 0009813) (Fig. 5, Supplementary Table S8), suggesting that expression of these genes is associated with SMF intensity but not with orientation. In the SMF-treated leaves, the common down-regulated DEGs were involved in the red (GO: 0010114) or far-red light signaling pathway (GO: 0010017) (Fig. 5, Supplementary Table S8), suggesting a crosstalk between SMF and red/far-red light signaling. Other biological processes were enriched uniquely in a particular treatment. For instance, plant-type cell wall organization (GO: 0009664), nitrate transport (GO: 0015706) and callose deposition in the phloem sieve plate (GO: 0080165) were enriched in the up-regulated N0R transcriptome, while photosynthesis related terms (GO: 0015979, GO: 0009658 and GO: 0010027) in the down-regulated N0R transcriptome (Fig. 5, Supplementary Table S8). Taken together, transcriptomic analysis uncovers biological processes regulated by SMF directions in roots and leaves.

### Main biological processes altered in N180-treated roots

To understand the mechanism by which SMF effect on root growth is lost when the root growth direction is kept oppositely to SMF orientation, we carefully looked at the 45 DEGs, in which 28 genes were up-regulated and 17 down-regulated, in N180-treated roots. Interestingly, about half (13 genes) of the up-regulated genes encode proteins involved in flavonoid biosynthesis and transportation (Supplementary Table S9), including a number of key genes in the flavonol biosynthetic pathway, such as two *PHENYLALANINE AMMONIA-LYASE* (*PAL1* and *PAL2*), *4-COUMARATE:COA LIGASE* (*4CL*), *FLAVONOIUD 3*′ *HYDROOXYLASE* (*TT7*), *FLAVONOL SYNTHASE* (*FLS1*), and several *UDP-GLYCOSYLTRANSFERASES* (*UGTs*). These results indicate that flavonoid biosynthesis in the root may be enhanced under the N180 condition. Flavonols have been reported to function as inhibitors for auxin transport^41,42^. Thus, it is likely that auxin basipetal transportation is affected in the N180-treated root. On the other side, we found that one of the 17 down-regulated genes was *YUC9*, a member of auxin biosynthetic *YUCCA* family genes that play a key role in local auxin production. Although the *yuc9* mutant has no obvious phenotype in primary root length under normal growth conditions, *YUC9* is reported to be required for aluminium stress-inhibited primary root growth via regulating auxin biosynthesis at the root transition zone^43^. In addition, there were 4 down-regulated genes encoding cell wall proteins, namely β-glycosidase 44 (BGLU44), β-galactosidase 2 (BGAL2), xyloglucan endotransglucosylase 24 (XTH24), and lipid transferase protein 3 (LTP3/PR14), suggesting that the relaxation of root cell wall is probably reduced by N180 treatment. Taken together, our data may reveal that intensity and direction of SMF are perceived differently and trigger differential transcriptional responses.

### Transcriptomic bases for N0-promoted root growth

To elucidate molecular mechanisms of the N0-promoted root growth at the transcriptional level, we narrowed down the DEGs with more than 2-fold (*p* < 0.05) changes. In comparison with control roots, a total of 420 DEGs, including 61 up-regulated and 359 down-regulated, were sorted out from the N0-treated roots (Supplementary Table S10). It is surprisingly that, among 359 down-regulated genes, 169 (47.75%) gene products were predicted to be in plastids. Classification of these plastid-localized proteins showed that 7 proteins, including sigma factor 1 (Sig1), Sig2 and Sig6, function in transcription, 19 in translation, 12 in RNA processing, 3 in protein import, 65 in photosynthesis, 31 in plastid metabolism, and 32 in unknown processes. These results suggest that N0 treatment suppresses chloroplast development in the roots. Among other 190 down-regulated genes, 16 genes encode transcription factors, 23 leucine-rich repeat protein kinases (RLK) and factors involved in signaling transduction, 7 in phytohormone metabolism, 31 in primary metabolism, 14 in secondary metabolism, 17 in transmembrane transport, and 20 in stress response (Supplementary Table S11). Among the transcription factor genes, we found two genes encoding GATA transcription factors, called *GATA, NITRATE-INDUCIBLE, CARBON METABOLISM-INVOLVED* (*GNC*) and *GNC-LIKE* (*GNL*), which play an important role in cytokinin-regulated chloroplast development in roots^44–46^. Recently, Kobayashi *et al*. (2017) reported that GNL acted at the point of convergence of cytokinin and auxin signaling in regulation of root greening. Since so many genes involved in chloroplast development are down-regulated by N0 treatment, we examined chloroplast development in the roots. Our results showed that N0 treatment led to decreases in the number of chloroplasts and chlorophyll content (Supplementary Fig. S4). Taken together, our data suggest that chloroplast development is inhibited in N0-treated roots. On the other side, among 61-upregulated genes, most (18 genes) encode functionally unknown proteins including 4 RLKs; next were three phytohormone metabolism-related genes, such as *GIBBERELLIC ACID 3-OXIDASE 2* (*GA3OX2*), *METHYL ESTERASE 1* (*MES1*) probably involved in hydrolyzing methyl salicylate (*MeSA*), and brassinosteroid *SULFOTRANSFERASE 4A* (*ST4A*); Functions of other genes were related to primary metabolism (6 genes), secondary metabolism (7 genes), nutrient transport (4 genes), stress response (7 genes) and other biological processes (8 genes). It is worth noting that GA3OX2 catalyzing the conversion of GA precursors to the bioactive forms is expressed in elongation cells, quiescent cells and columella cells of the primary root tip^47^. In addition, several auxin responsive genes were regulated by SMF treatments (Supplementary Table S12). Taken together, our data indicate that N0 treatment leads to changes in levels of some phytohormones in roots.

### N0-promoted root growth is regulated by auxin signaling

**Figure 6:**
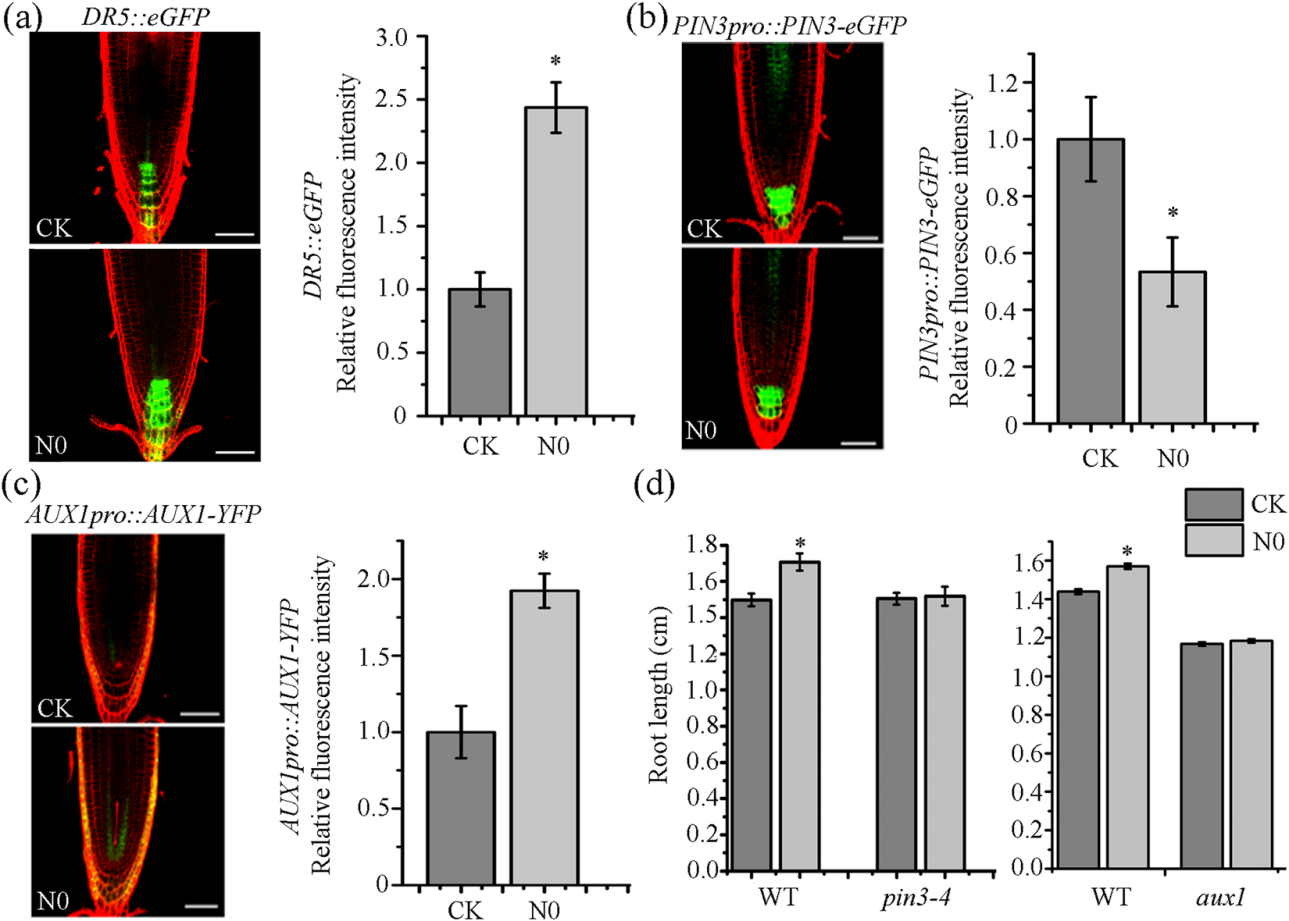
N0-promoted root growth is regulated by auxin signaling. (**a**) Auxin concentrations in N0-treated and control root tip cells were analyzed using the auxin-responsive reporter *DR5::eGFP* line. The cellular expression pattern of GFP in the root is shown in the left panel, whereas quantification of GFP signal is shown in the right panel. (**b**) Expression levels of PIN3 in the roots of *PIN3pro::PIN3:eGFP* seedlings treated with or without N0. (**c**) Expression levels of AUX1 in roots of *AUX1pro::AUX1:YFP* seedlings treated with or without N0. **(d**) Comparison of primary root length between WT and *pin3-4* or *aux1* seedlings treated with or without N0. All data are presented mean ± SE (n > 60). Three biological experiments were performed, and similar results were obtained. *Indicates a significant difference at *P* < 0.05 (Student *t*-test) between N0 treatment and the control.

The data derived from the above transcriptomic analysis prompted us to test the hypothesis whether N0 treatment leads to an increase of auxin content in the root meristem. To do this, we employed the auxin-responsive reporter *DR5::eGFP* line to examine N0 effects on the auxin level and its cellular distribution^48^. The results showed that GFP signal in the N0-treated root tip was about 2.5-fold of that in the control (Fig. 6a), but the pattern of GFP signals distributed in quiescent cells, columella initials and columella cells was not significantly altered between N0 treatment and control (Fig. 6a). Since auxin accumulation in the primary root tip is regulated by both auxin efflux (PINs) and influx (AUX1/LAXs) carriers, we reasoned that expression levels of these carriers might be altered by N0 treatment. To test this hypothesis, we first studied the effect of N0 on the accumulation and polarization of PINs using the native promoter-driven expression of the GFP-fusion carries. These lines include *PIN1pro::PIN1-GFP*, *PIN2pro::PIN2-GFP*, *PIN3pro::PIN3-GFP*, and *PIN7pro::PIN7-GFP*^49^. In general, we found that N0 treatment had no clear impact on cell-specific expression patterns and the polarity of these PINs. As previously reported, PIN1-GFP was mainly localized at the basal side of the stellar and endodermal cells (Supplementary Fig. S5a), whereas PIN2-GFP was expressed in the apical side of epidermal, lateral root cap and cortical cells (Supplemetnal Fig. 5b), PIN3-GFP strongly in columella cells (Fig. 6b), and PIN7-GFP mainly in columella and provascular cells (Supplementary Fig. S5c). With regard to the expression level of these PINs, N0 treatment led to a significant reduction of PIN3-GFP in columella cells to 45% of the control, but had no obvious impact on the levels of other PINs (Fig. 6b). These results indicate that PIN3 expression is specifically down-regulated by N0 treatment. To further confirm the positive role of PIN3 in N0-stimulated root growth, we grew *Arabidopsis pin3-4* and *pin7-2* mutants under the N0 condition. As shown in Fig. 6d and Supplemetary Fig. 6c, N0 treatment significantly promoted root growth of both WT and *pin7-2* seedlings, but had no effect on root length of *pin3-4* seedlings. Thus, our evidence supports that PIN3 is involved in plant responses to SMF stimuli.

We also examined N0 effect on the expression level of the auxin import carrier AUX1 using the *AUX1pro::AUX1-YFP* marker line. In the root meristem of the control seedlings, AUX1-GFP was localized in the columella cell, lateral root cap, epidermis and stele (Fig. 6c). However, N0 treatment markedly enhanced AUX1-GFP signal in root meristem cells. Consistently, quantification of GFP signal intensity showed that N0-treated meristem cells accumulated about 2-fold AUX1 of the SMF-untreated cells. We also analyzed the response of loss-of-function *aux1* mutants to N0 treatment, and found that *AUX1* mutations led to no difference in root length between N0-treated and untreated seedlings (Fig. 6d). These results indicate that AUX1 also contributes to a higher level of auxin in N0-treated roots.

Taken together, our data suggest that PIN3 and AUX1 function in a coordinated manner to enhance auxin basipatel flux and reduce auxin transport away from the root apex, ultimately leading to an increase in auxin content in the N0-treated root tip cells.

### Photoreceptors CRYs are involved in SMF-promoted root growth

**Figure 7:**
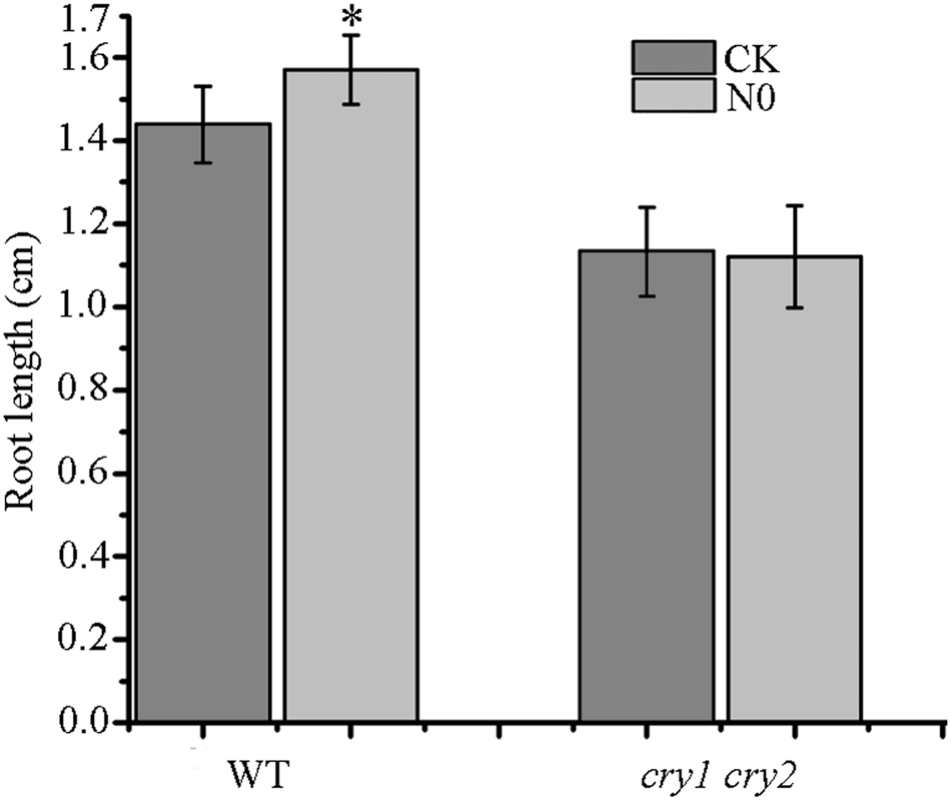
Comparison of primary root length between WT and *cry1 cry2* seedlings treated with or without N0. Three biological experiments were performed, and similar results were obtained. *Indicates a significant difference at *P* < 0.05 (Student *t*-test) be*t*ween N0 treatment and the control.

Considering that CRYs have been demonstrated to mediate plant responses to SMF^6,7,18,21,22,24,50,51^ and to directly interact with transcription regulators auxin/indole-3-acetic acid proteins (AUX/IAA) in auxin signaling^5,10^, we examined whether CRYs are involved in SMF-promoted root growth. As shown in Fig. 7, the *cry1 cry2* double mutant produced significant shorter roots than WT under the local geomatical field (CK), indicating that CRYs are positive regulators for primary root growth. Root length were very similar between *cry1 cry2* seedlings treated with and without N0, while that of WT was significantly longer in N0 than in CK. These results suggest that CRYs play a role in root responses to SMF.

## Discussion

In this study, we systematically investigated biological effects of SMF intensity and direction generated from the permanent sintered magnet. Under our experimental conditions, we discovered that effects of SMF on *Arabidopsis* young seedlings depend not only on its intensity and direction but also on tissues. Root growth, but not leaf and hypocotyl growth, is significantly enhanced by all treatments of 600 mT SMF except for N180. Thus, the root is the most sensitive organ in the *Arabidopsis* seedling in response to SMF stimuli. SMF treatments promote cell division of root meristems, but have no impact on cell size in the mature zone. We also provided several lines of physiological and molecular evidence to support the SMF-promoted root growth. For example, the level of auxin increases in the N0-treated root tip via enhanced expression of auxin influx transporter AUX1 and decreased expression of auxin efflux transporter PIN3 (Fig. 6); consistently, high throughput transcriptomic assays revealed that N0 treatment significantly downregulated expression of genes functioning in chloroplast development in roots probably via auxin signaling. Additionally, our genetic data showed that CRYs are involved in root responses to SMF. Thus, the *Arabidopsis* primary root could be a good model to study molecular mechanisms by which plants sense magnetic fields, and transduce the signal into the cellular biological processes in the future.

To date, a large body of papers have demonstrated biological effects of magnetic field intensities higher than the GMF level. Most data showed that a moderate SMF intensity from 0.1 to 1000 mT can promote seed germination and subsequent seedling growth whatever dry or imbibed seeds are magnetically treated^11,13^. Here, we did not observe significant effects of 300 mT SMF on *Arabidopsis* seedling growth compared to the control. The different results could be related to our experimental design and plant growth conditions. We selected the 3-day-old seedlings with the same primary root length as starting materials for SMF treatment in order to exclude the positive effect of SMF on seed germination. It is possible that the SMF intensity required for promoting root growth is higher than that for seed germination. Additionally, seedlings were grown on half MS media supplemented with 1% sugar in our study, while in most cases seedlings were grown in soil. We speculate that the limited effect of 300 mT SMF on plant growth is probably masked by rich nutrition conditions, since SMF-promoted root growth is correlated with root absorption of nutrients and water^11,52^. The interaction between culture media and MF effects was more apparent in the tissue culture system^53^. A number of reports indicated that SMF had stress-protecting roles in various abiotic stresses^54,55^. Mung bean seedlings treated with 600 mT MF had an increased resistance to cadmium stress probably via NO signaling^56^. Thus, we cannot exclude the possible role of 300 mT SMT in stress conditions. It will be important for our understanding of biological significance of SMF in plant adaptation to various environment conditions.

In most publications, little information is provided about derections of a magnetic field and plant growth. In the present study, the best biological effect of 600 mT SMF on primary root growth is observed when the root tip is orientated straightly to the south pole of the magnet while the weakest when the root tip is orientated straightly to the north pole (Fig. 1), which is generally in agreement with previous reports^57^. Roots of barley, maize, radish, vetch and cucumber seedlings grew more rapidly when they were oriented to the magnetic south pole of a magnet^58^. The magnetic field direction could become an important factor to affect biological processes particularly when plants are put in a non-homogeneous magnetic field or in the field with magnetic gradients, because magnetic gradients have been reported to influence gravitropic sensing in stems and roots^57^. Consistently, biological effects of SMF (250 or 500 mT) on root growth disappeared when *in vitro* plagiotropic hairy roots were used in experiments^59^. This characteristic of SMF effects on root growth suggests a close correlation between magnetic fields and gravity. Actually, it has been demonstrated that magnetic fields can determine the displacement of amyloplasts, indicative of a partially overlapping mechanism with gravitropism^60^. Interestingly, Kato (1988) reported quite similar results using a *Zea mays* cultivar Golden Cross Bantam 70, which is able to elongate horizontally without a geotropic response under dark conditions^61^. When the direction of root growth was parallel with or perpendicular to the direction of the 500 mT MF (produced by a superconductive coil), the growth rate of roots was 27 and 15% higher than the control treatment (1 mT), respectively. Different from our data, they also observed MF-treated roots had 22% higher growth rate than the control treatment, when the direction of MF was opposite to the direction of root growth. Such a difference could be caused by different types of magnets and plant species used. Recently, differential effects of magnetic field directions were also found in human cell lines^62^, indicative of conservative roles of magnetic field directions in various biological systems.

In this study, the significant root growth phenotype of N0-treated young seedlings provided us an excellent opportunity to dissect molecular processes determining the SMF action. In migratory animals, it is well recognized that magnetic fields are perceived by CRYs, which form a pair of radicals with correlated spins when exposed to blue light or by the light-independent dark reoxidation of the flavin cofactor^50^. In plants, although the role of CRYs in responses to magnetic fields had been controversially reported^50,51^, recent publications unanimously support that CRYs are required for magnetic field-induced biological processes^6,7,22,24^. Here, our data also reveal that CRY are involved in SMF-promoted *Arabidopsis* root growth. *Arabidopsis* CRYs are known to regulate a series of biological processes for plant growth and development, such as photomorphogenesis, stomatal development, circadian rhythm and flowering, via multiple downstream effectors. To date, however, it remains unknown how CRYs mediate SMF-triggered plant phenotypes. Our transcriptomic, molecular and genetic analyses indicated that auxin plays an important role in SMF-promoted root growth. With regard to the crosstalk between CRY and auxin signaling pathways, photoactivated CRY1 interacts directly with and subsequently stabilizes the master regulators AUX/IAA proteins in auxin signaling, leading to repression of auxin-responsive gene expression^5^. This regulation of AUX/IAA proteins by CRY1 is well fit for elucidating molecular mechanism on hypocotyl elongation which is inhibited by CRY1 but is promoted by auxin. In contrast, CRYs, like auxin, function as a positive regulator for SMF-promoted root growth. Based on the evidence derived from this work, we propose that SMF-triggered changes in PIN3 and AUX1 expression is post-transcriptionally regulated, because there was no significant difference in mRNA levels of *PIN3* and *AUX1* between N0-treated and untreated roots (Supplementary Fig. S5). Thus, it will be interesting to investigate how CRY signaling is linked to the post-transcriptional regulation of these auxin acrriers in root responses to SMF.

The important role of auxin in plant responses to magnetic fields has been demonstrated in the delayed flowering phenotype of *Arabidopsis* plants in NNMF^5^. Simultaneously, Xu *et al*. (2018) detected a significantly higher IAA level in NNMF-treated roots than the local geomagnetic field control, but the corresponding phenotype of the roots was not mentioned^5^. In this study, our data from transcriptomic analysis also indirectly supported that auxin levels are altered in SMF treated roots. In N0-treated roots, about half of the 359 downregulated genes with more than two-fold changes compared to the control function in chloroplasts (Supplementary Fig. S4 and Supplementary Table S9), suggesting that chloroplast development is suppressed by 600 mT SMF. In general, root cells should not develop chloroplasts without light signal or other external stimuli, but can partially turn green in light^44,63^. Chloroplast development in roots is actually repressed by auxin signaling via suppressing expression of key transcriptional factors, such as GNC and GNL^46,63^. In agreement with these published results, our data indeed revealed that expression of *GNC* and *GNL* was significantly downregulated by 600 mT SMF, and subsequently the degree of chloroplast development in the SMF-treated roots was lower than in the control (Supplementary Fig. S4). However, such a change in the gene expression profile was not observed in N180-treated roots, which had no significant difference in root length compared to the control, suggesting that in N180 treatment the intensity and direction of SMF function antagonistically. Interestingly, a number of genes upregulated in N180-treated roots function in the biosynthesis of flavonols, which are inhibitors for auxin basipetal transportation, ultimately leading to no change of the auxin level in the root tip cells^64^. Taken together, our data suggest that biological effects of SMF on plant growth are highly associated with auxin.

It has been well documented that auxin plays a crucial role in mediating the plant response to gravity. In gravity perception, PIN3 and PIN7 first relocalize to the basal side of columella cells to mediate auxin redistribution^48^, and then AUX1 and PIN2 are responsible to transport auxin to epidermal cells in the elongation zone through the lateral root cap^29^, ultimately resulting in differential accumulation of auxin between the upper and lower parts of the root tip. Recently, Zhang *et al*. (2019) reported that auxin can regulate the biosynthesis of starch granules within the root apex^65^, suggesting an important role of auxin in the perception of the gravity signal. Thus, addressing the question whether root gravitropism is affected by SMF will provide new insights into molecular mechanisms by which SMF modulates auxin distribution in the future.

## Materials and Methods

### Plant materials and growth conditions

The plant materials used in this study were in the Columbia-0 ecotype background, including *aux1*, *pin3-4* and *pin7-2* mutants^49^, *AUX1pro::AUX1-YFP*, *PIN1pro::PIN1-GFP*, *PIN2pro::PIN2-GFP*, *PIN3pro::PIN3-eGFP*, *PIN7pro::PIN7-GFP*, *CYCB1;1::GUS* and *DR5::eGFP* transgenic plants^49^. The surface-sterilized seeds were treated at 4 °C in the dark for 2 days and then germinated on the half-strength Murashige and Skoog (MS) medium (pH 5.7) (Sigma-Aldrich) containing 0.7% phytogel and 1% sucrose at 20 °C with a light intensity of 100 μmol m^−2^ s^−1^ in long-day conditions (16 h light/8 h darkness). To exclude the effect of SMF on seed germination rate, the uniform 3-day-old seedlings were transferred to the fresh media. Seven-day-old seedlings were sampled for various assays.

### SMF and local environment control

The permanent sintered magnets Nd-Fe-B banded N52, which contains PrNd (31 wt.%), B (1 wt.%), Fe (67.5 wt.%) and other (0.5 wt.%) (Hangzhou Permanent Magnet Group Co. Ltd, Xiaoshan, Hangzhou, P.R. China, www.china-hpmg.com), were used in this study. These magnets were cubes with each side length 10 cm with the surface magnetic strength of 600 mT on average. The SMF treatments were classified into four types depending on the parallel (N0), opposite (N180), and angles perpendicular (N90) orientation of a magnet to the root growth (gravity) direction (Fig. 1a). Perpendicular treatments were further divided into three subtypes depending on seedlings close to the north pole (N90N), the south pole (N90S) or in the side between the north and south poles (N90M) (Supplementary Fig. S1). Petri dishes were putted closely to each side of a magnet for the magnetic treatment or an iron cube for a control. The local geomagnetic field intensity is approximately 0.05 mT.

### Measurement of primary root length and meristem size

To quantify primary root length, we firstly scanned seedlings in a petri dish with the scanner of EPSON Perfection 4490 PHOTO (Seiko Epson Corporation, Japan), then measured primary root length with image J software. For measurement of root meristem size, meristem cell number and cortical cell length in the elongated zone, seedlings were stained with 1 mg/L propidium iodide (PI, Sigma P4170) for 7 min, and then washed with deionized water for 5 min. Olympus FV1000 was used to excite PI fluorescence with 30% maximum laser power at 535 nm, and the image of a root was recorded. The root meristem zone was defined the region from the quient cell (QC) to the first elongated cell. The number of root meristem cells were counted using the cortical cells in the root meristem zone. The size and cell number of root meristems, and cortical cell length of root elongated zone were measured by software ImageJ. All experiments were carried out for 3 to 4 biological replicates, and at least 60 seedlings were analyzed in each replicate. The data were statistically analyzed by Student’s *t*-test.

### Histochemical staining and microscopic observation

For GUS staining, 7-day-old seedlings treated with or without a magnet were harvested to stain immediately in the staining buffer (1% dimethylformamide, 0.1 mM K_3_[Fe(CN_6_)], 0.1 mM K_4_[Fe(CN_6_)], 1 mM EDTA 50 mM Na_2_HPO_4_, 50 mM NaH_2_PO_4_, 1 mg/mL X-Gluc (2 mM 5-bromo-4-chloro-3-indolylglucuronide, pH 7.0) at 37 °C for 24 hours, and cleared by incubation in 75% ethanol. Plants were photographed using OLYMPUS SZX16 (Olympus Corporation, Japan). Fluorescent signals including GFP, YFP and propidium iodide (PI) were recorded by a confocal laser scanning microscope (Fluoview FV3000, Olympus Corporation, Japan) GFP, YFP, and PI were excited at 488, 514, and 543 nm, and emitted light collected at 510–550, 530–570, and 600–640 nm, respectively. Seedlings were stained with 10 mg/mL of PI for 8 min and washed three times in water. Approximately 40 images were grabbed using Olympus FV1000 and the fluorescence intensity was measured using image J software according to the method^66^ for each replicate, and at least three independent experiments were performed. The chlorophyll contents were determined as described previously^67^.

### Analyses of gene expression and transcriptomes

Total RNAs were extracted from samples with the RNAgents Total RNA Isolation System (Promega, Z5110), and then treated with the DNA-free Kit (Applied Biosystems, AM1906). The first-strand cDNA was synthesized using oligo (dT) 18 from 1 μg RNA with a Reverse Transcription System (Promega, A3500). Quantitative real-time PCR was performed with gene-specific primers using a total volume of 20 μl of SYBR Premix Ex Taq (TaKaRa, RR420A), and monitored on QuantStudio 3 (Thermo Fisher Scientific, America) according to the method^68^. *ACTIN2* was used as an internal control. The primers used were listed in Supplementary Table S12.

Transcriptomes for roots and leaves of 7-day-old seedlings were analyzed by Shanghai Biotechnology Corporation, China. The quality control of the raw data was examined using the FastQC tool (http://www.bioinformatics.babraham.ac.uk/projects/fastqc/). The sequenced raw reads were processed to obtain clean reads using the following strategy: (i) removal of reads with adaptor contamination; (ii) removal of low quality reads whose average quality was less than Q20; and (iii) removal of the reads with a final length of less than 50 bp. Then, the high-quality clean paired-end reads were mapped against the *Arabidopsis* genome provided by Ensembl database (version TAIR 10) using Bowtie2 and TopHat2^69^. The expression levels were calculated using the HTSeq program (version 0.9.1)^70^, which employs unique reads for the estimation of gene expression and eliminates the multimapped reads. Pearson correlation coefficient of gene expression in every three replications was calculated and satisfied the condition of R^2^ > 0.85. Differential expression analysis between conditions was assessed using the DESeq2 method^71^, which applies a differential negative binomial distribution for the statistical significance. Up- or down-regulation of a gene was considered to have occurred when the *P* value adjusted by Benjamini-Hpchberg FDR was <0.05^72^ and fold changes were >1.5. Gene ontology (GO) and analyses of the genes with differential expression were performed with using DAVID Bioinformatics Resources 6.8^73^.

## Supplementary information

Supplementary information

Supplementary dataset
