# Supplementary material for: Static magnetic field regulates Arabidopsis root growth via auxin signaling
Source: Sci Rep. 2019 Oct 7;9:14384. doi: 10.1038/s41598-019-50970-y (PMC6779896; doi:10.1038/s41598-019-50970-y)
Supplement: Supplementary file 1 — Supplementary information [file 41598_2019_50970_MOESM1_ESM.pdf]

Title

Static magnetic field regulates *Arabidopsis* root growth via auxin signaling

Authors

Yue Jin[1#], Wei Guo[1#], Xupeng Hu[2#], Mengmeng Liu[1], Xiang Xu[1], Fenhong Hu[1], Lan[3], Chenkai Lv[3], Yanwen Fang[4], Mengyu Liu[4], Tieliu Shi[3], Shisong Ma[5], Zhicai Fang[4], Jirong Huang[1*]

[1]Shanghai Key Laboratory of Plant Molecular Sciences, College of Life Sciences, Shanghai Normal University, Shanghai 200234, China

[2]Institute of Plant Physiology and Ecology, Chinese Academy of Sciences, Shanghai 200032, China

[3]Shanghai Key Laboratory of Regulatory Biology, School of Life Sciences, East China Normal University, Shanghai, 200241, China

[4]Heye Health Industrial Research Institute of Zhejiang Heye Health Technology, Anji Zhejiang 313300, China

[5]School of Life Sciences, University of Science and Technology of China, Hefei 230026, China

[#] These authors contributed equally to this work.

* Corresponding author: e-mail, huangjr@shnu.edu.cn; fax, +86-21-64322033

Supporting Information

Supporting information includes 6 figures and 12 tables.

Supplementary Figure S1 Effects of three N90 treatments with the intensity of 600 mT SMF on root growth.

Supplementary Figure S2 Effects of N0 and N180 treatment with 600 mT SMF on the size of cotyledons and hypocotyls of 7-day-old seedlings.

Supplementary Figure S3 Effects of 300 mT SMF on primary root growth of 7-day-old seedlings.

Supplementary Figure S4 Chloroplast development in roots of 7-day-old seedlings is suppressed by SMF treatment.

Supplementary Figure S5 Analysis of effects of N0 treatment on expression patterns of auxin efflux transporters using transgenic lines.

Supplementary Figure S6 Quantification of root meristem size, meristematic cortex cell number of WT and *pin3-4*, and root length of *pin7-2* seedlings treated with or without N0.

Supplementary Table S1 An overview of the RNA sequencing quality of all samples.

Supplementary Table S2 Read number for all genes under the various SMF conditions.

Supplementary Table S3 Correlations of gene expression levels between replicates.

Supplementary Table S4 Average fold changes in all genes under the various SMF conditions.

Supplementary Table S5 Genes significantly up- or down-regulated by SMF treatments.

Supplementary Table S6 Genes significantly regulated by SMF in more than one treatment.

Supplementary Table S7 UDP glycosyltransferase genes (UGTs) up- or down-regulated by SMF.

Supplementary Table S8 GO analysis of biological processes using DEGs in leaves (L) and roots (R).

Supplementary Table S9 Functional classification of DEGs from N180-treated roots.

Supplementary Table S10 Functional classification of DEGs from N0-treated roots.

Supplementary Table S11 Auxin-related genes regulated by SMF.

Supplementary Table S12 Primers used in this study.

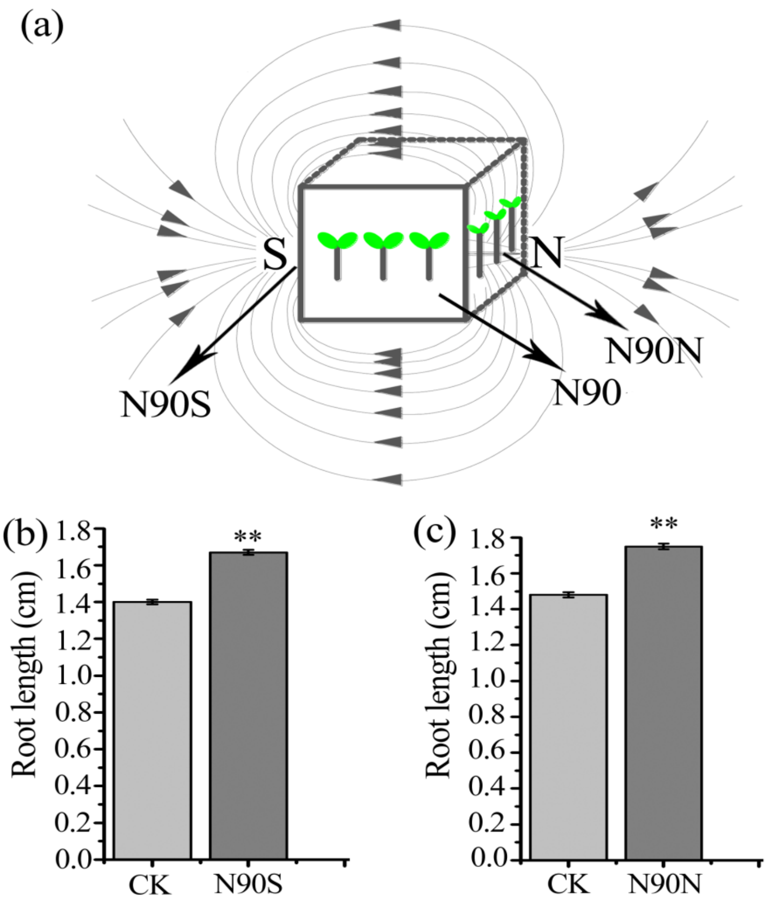

Supplementary Figure S1 Effects of three N90 treatments with the intensity of 600 mT SMF on root growth. (a) A diagram shows the three N90 treatments (N90N, N90 and N90S). Plates are put to the S and N pole of a magnet in N90S and N90N treatments, respectively, whereas in N90 treatment to the surface between the two poles. (b, c) Primary root length of 7-day-old seedlings treated with N90S (b) and N90N (c). The data are shown means ± SE (n > 60). ** indicate significant differences at $P < 0.01$ (Student *t*-test). At least three independent biological repeats were conducted.

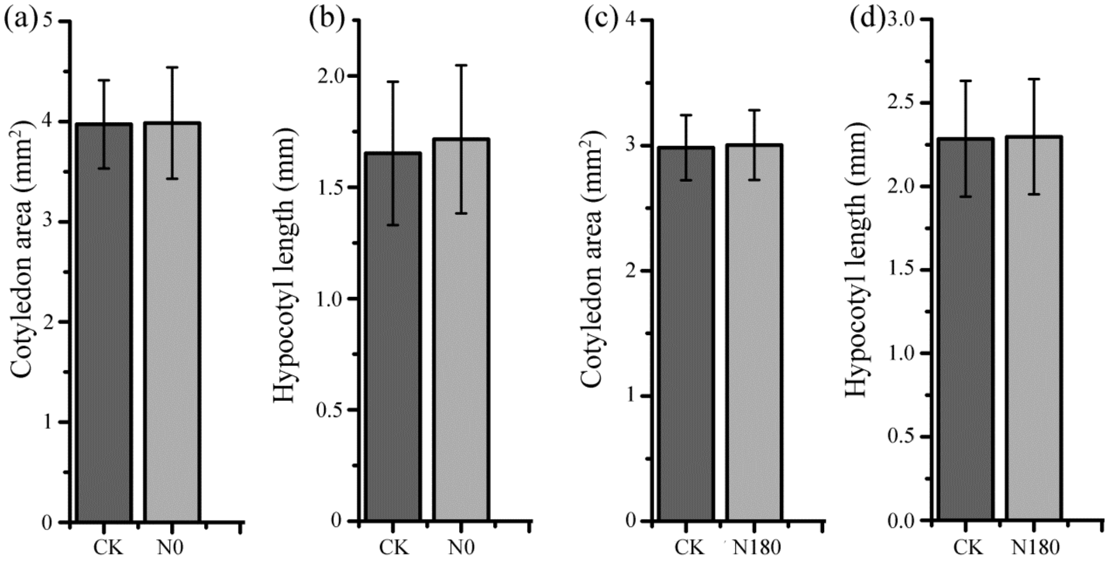

Supplementary Figure S2 Effects of N0 and N180 treatment with 600 mT SMF on the size of cotyledons and hypocotyls of 7-day-old seedlings. (a-d) Comparison of cotyledon areas (a, c) and hypocotyl length (b, d) between N0 (a, b)- or N180 (c, d)-treated and the control. Results are shown mean ± SE (n > 60), and were repeated independently more than 3 times.

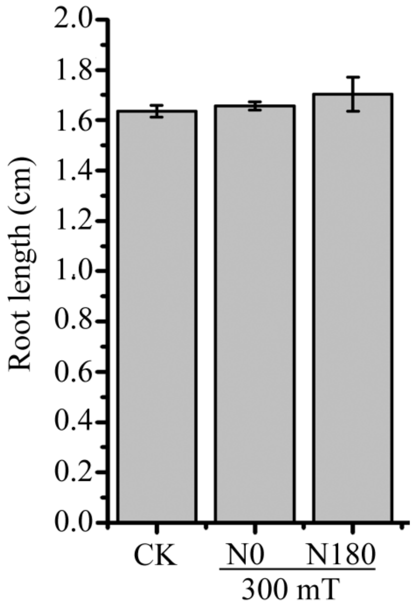

Supplementary Figure S3 Effects of 300 mT SMF on primary root growth of 7-day-old seedlings. Comparison of root length between N0 (a) or N180 (b) treatment and the control. Data are presented mean ± SD (n > 60). The results were repeated independently more than 3 times.

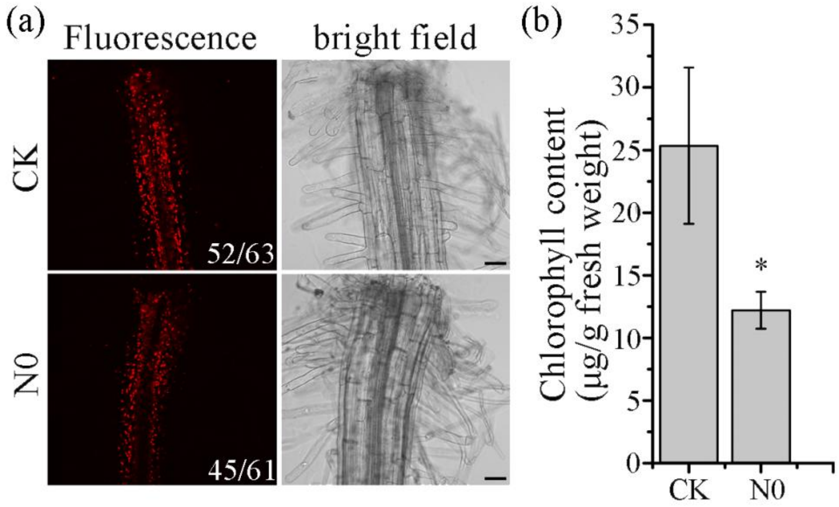

Supplementary Figure S4 Chloroplast development in roots of 7-day-old seedlings is suppressed by SMF treatment. (a) Autofluorescence of chloroplasts was recorded in the upper root part close to the connection of the hypocotyl. (b) Chlorophyll content in the roots. The data are shown means ± SE (n > 60), and were repeated independently more than 3 times. * indicates a significant difference between the treatment and control at $P < 0.01$ (Student *t*-test).

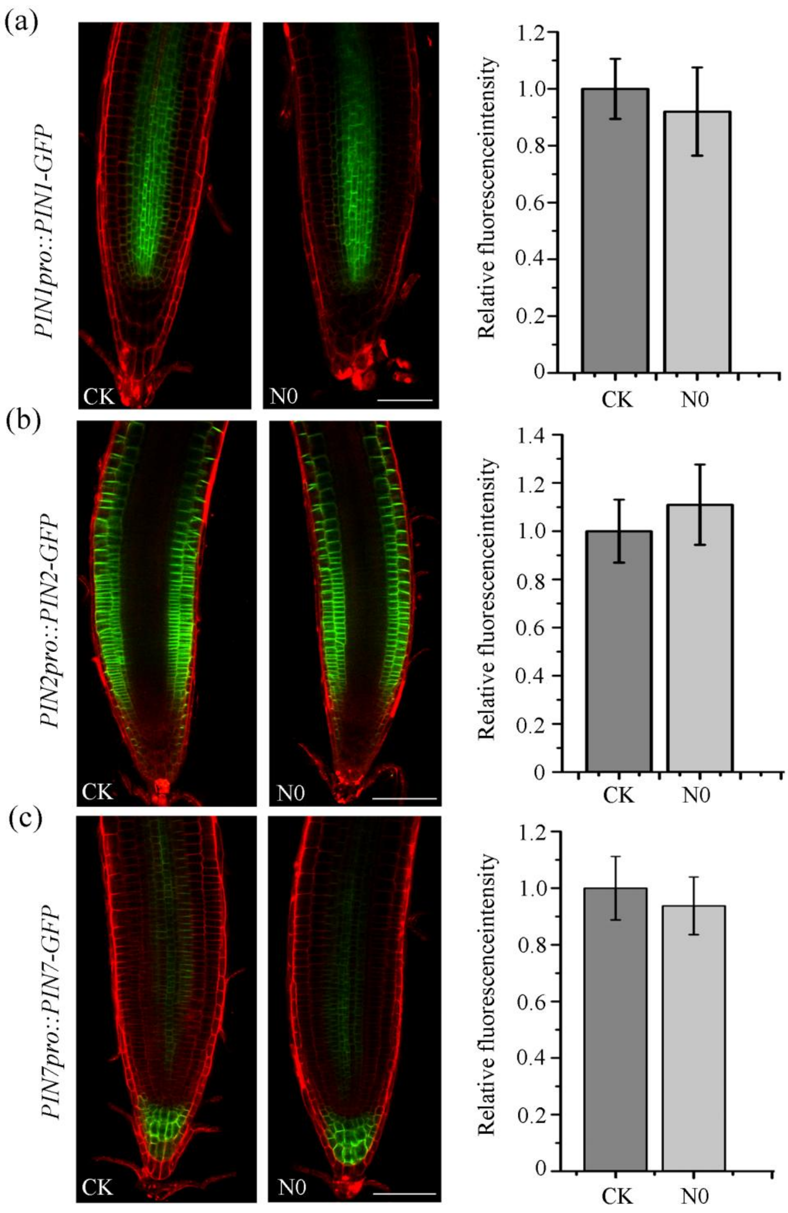

Supplementary Figure S5 Analysis of effects of N0 treatment on expression patterns of auxin efflux transporters using transgenic lines. (a-c) Expression levels of PIN1 (a), PIN2 (b) and PIN7 (c) in the roots of *PIN1pro::PIN1-GFP*, *PIN2pro::PIN2-GFP* and *PIN7pro::PIN7-GFP* seedlings under SMF and control conditions, respectively. Left panels show fluorescent signals in the root tip of 7-day-old seedlings, whereas right panels show relative fluorescence intensity. The data are shown mean ± SE (n > 40), and the Student *t*-test analysis showed no significant differences between the treatment and control.

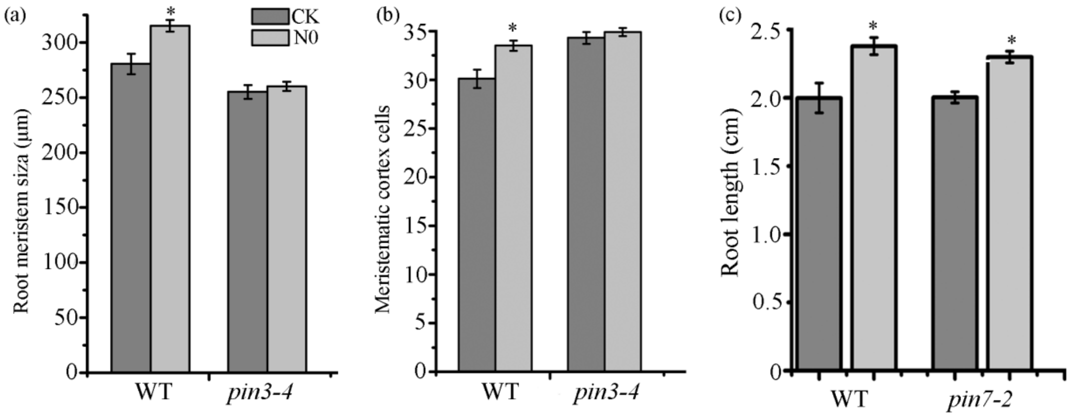

Supplementary Figure S6 Quantification of root meristem size, meristematic cortex cell number of WT and *pin3-4*, and root length of *pin7-2* seedlings treated with or without N0. Three biological experiments were performed, and similar results were obtained. * indicates a significant difference at $P < 0.05$ (Student *t*-test) between N0 treatment and the control.
